# A148 *NOD2* LIGAND AND PEPTIDOGLYCAN HYDROLASE GENE ABUNDANCES ARE ALTERED IN PRE-CROHN’S DISEASE

**DOI:** 10.1093/jcag/gwae059.148

**Published:** 2025-02-10

**Authors:** M Ren, O de Sa, Q LI, B Bharali, K Mu, W Turpin, K Croitoru, o.n behalf of the CCC-GEM Research Consortium, D Philpott

**Affiliations:** University of Toronto, Toronto, ON, Canada; University of Toronto, Toronto, ON, Canada; Lunenfeld-Tanenbaum Research Institute, Toronto, ON, Canada; Lunenfeld-Tanenbaum Research Institute, Toronto, ON, Canada; University of Toronto, Toronto, ON, Canada; University of Toronto, Toronto, ON, Canada; University of Toronto, Toronto, ON, Canada; full list available at www.gemproject.ca, Toronto, ON, Canada; University of Toronto, Toronto, ON, Canada

## Abstract

**Background:**

Crohn’s disease (CD) is a chronic disorder of unknown etiology. Genome-wide association studies have revealed that *NOD2* loss of function mutations are an important gene contributor to CD pathogenesis. *NOD2* is a pattern recognition receptor that responds to phosphorylated muramyl-dipeptide (MDP), whose production from peptidoglycan (PGN) is dependent on the expression of specific microbial hydrolases in the lumen. While a variety of studies have shown that reductions in *NOD2* ligand and corresponding hydrolases, particularly DL-endopeptidases, occur in active CD or as a consequence of inflammation, which may play a role in sustaining disease, the interplay between *NOD2* ligands and PGN-hydrolases pre-disease is unknown.

**Aims:**

We investigated whether stool *NOD2* ligand concentration is altered in pre-CD individuals, and the associations of PGN-hydrolase abundances with *NOD2* ligand and with other pre-disease biomarkers.

**Methods:**

Fecal samples were collected from healthy first-degree relatives of CD patients who were followed prospectively as part of the CCC-GEM project nested case control cohort (n = 91 pre-CD, n = 242 matched controls, median time to diagnosis 3.2 years). Fecal calprotectin (FCP), urinary fractional excretion ratio of lactulose to mannitol (LMR), and C-reactive protein (CRP) from serum were measured. PGN-hydrolase gene abundances was assessed by shotgun sequencing of stool samples. *NOD2* ligand abundance was measured by incubating diluted stool supernatant with human *NOD2* reporter HEK293 cells overnight then measuring OD_640_. A database of PGN-hydrolase clusters was generated by sequentially clustering with MMseqs2 based on sequence identity, then with Foldseek based on the AlphaFold Protein Structure Database.

**Results:**

*NOD2* ligand concentration was significantly reduced in pre-CD compared to matched controls (*p* < 0.01). Although several PGN-hydrolase clusters were negatively associated with *NOD2* ligand, the relative abundance of one DL-endopeptidase cluster expressed by the *Firmicutes* phylum was significantly positively associated with *NOD2* ligand abundance (*q* < 0.05). A DD-carboxypeptidase cluster, an amidase cluster, and a muramidase cluster were each negatively associated with future diagnosis of CD (*q* < 0.05). While many clusters were positively and negatively associated with FCP and CRP, one DD-carboxypeptidase cluster was positively associated with LMR (gut barrier permeability, *q* < 0.05).

**Conclusions:**

These results show that *NOD2* ligand concentration and the PGN-hydrolases that produce it are altered in pre-CD stool, suggesting that this may contribute to disease onset, however further investigation is needed.

**Funding Agencies:**

CCC, CIHRHelmsley Charitable Trust, Mount Sinai Hospital

